# Optimal timing for hepatic cyst intervention: Predictive factors for complete resolution after ethanol sclerotherapy

**DOI:** 10.1097/eus.0000000000000208

**Published:** 2026-08-18

**Authors:** Yoonchan Lee, Dong-Wan Seo, Sung Hyun Cho, Gunn Huh, Dongwook Oh, Tae Jun Song

**Affiliations:** 1 Department of Gastroenterology, Asan Medical Center, University of Ulsan College of Medicine, Seoul, Republic of Korea; 2 Division of Gastroenterology, Department of Medicine, Faculty of Medicine, Chulalongkorn University, Bangkok, Thailand.

**Keywords:** endosonography, liver cysts, sclerotherapy

## Abstract

**Background and Objectives:**

The optimal timing of ethanol sclerotherapy and predictors of complete resolution (CR) in patients with symptomatic hepatic cysts remain unclear. This study aimed to identify clinical and procedural predictors of CR and to determine a volume-based threshold that may guide patient selection.

**Methods:**

We retrospectively analyzed 307 patients who underwent percutaneous catheter drainage–guided or EUS-guided ethanol sclerotherapy between 2003 and 2023. CR was defined as <5% of baseline cyst volume. Receiver operating characteristic analysis identified a volume threshold for CR, and multivariable logistic regression was used to determine independent predictors.

**Results:**

CR was achieved in 54.4% of patients during a mean follow-up of 24.7 ± 21.1 months. An initial cyst volume of 233 mL (≈7–8 cm in diameter) was identified by receiver operating characteristic analysis as a threshold associated with CR (area under the curve 0.626; sensitivity 50.9%; specificity 75.0%). CR rates were significantly higher in cysts <233 mL than in larger cysts (70.6% *vs.* 44.1%, *P* < 0.001). In multivariable analysis, cyst volume ≥233 mL (adjusted odds ratio [aOR] 0.27, 95% confidence interval [CI] 0.15–0.48) and elevated alanine aminotransferase >30 U/L (aOR 0.16, 95% CI 0.05–0.50) were independently associated with nonresolution, as was diabetes mellitus (aOR 0.12, 95% CI 0.02–0.62), a finding requiring external validation given the small diabetic subgroup (*n* = 11). Treatment modality (EUS *vs*. percutaneous catheter drainage) was not associated with treatment success (*P* = 0.678).

**Conclusion:**

Initial hepatic cyst volume is an important factor influencing treatment outcome. The 233 mL threshold may inform the timing of intervention alongside symptoms and patient-specific factors.

## INTRODUCTION

Hepatic cysts are common benign liver lesions, typically asymptomatic and discovered incidentally during imaging studies performed for unrelated reasons.^[1,2]^ However, some cysts enlarge over time, leading to abdominal discomfort, early satiety, nausea, or complications such as rupture, hemorrhage, or infection.^[3,4]^ In such cases, intervention is warranted. Yet, the optimal timing and modality of treatment remain subjects of clinical debate.

Asymptomatic cysts are generally managed conservatively,^[5]^ while symptomatic cases may be treated with options ranging from percutaneous catheter drainage (PCD) and sclerotherapy to laparoscopic fenestration or surgical resection.^[6,7]^ Among these, PCD and EUS-guided ethanol sclerotherapy have emerged as minimally invasive and effective alternatives for patients unsuitable for surgery.^[8,9]^

Despite their efficacy, the threshold for initiating intervention is not clearly defined in existing guidelines.^[10]^ In practice, many clinicians defer treatment until the cyst becomes markedly large or severely symptomatic. However, emerging evidence suggests that earlier intervention may improve clinical outcomes.^[11]^ Larger cysts are associated with poorer drainage response, higher recurrence rates, and increased procedural burden.^[12]^ They may also compress adjacent structures, complicating the disease course with obstructive symptoms or infection.

Some studies have proposed volume-based criteria to guide intervention, suggesting that cysts smaller than 500 mL or less than 10 cm in diameter are more likely to achieve complete resolution (CR) after a single treatment session.^[13]^ This observation is supported by a 2017 systematic review, which reported proportional volume reductions ranging from 76% to 100% and symptom relief in 72%–100% of patients after aspiration sclerotherapy, particularly among those with smaller or solitary cysts.^[14]^ However, the review also noted considerable heterogeneity in sclerotherapy protocols and a high risk of bias across included studies, emphasizing the need for robust real-world evidence.

This study aims to evaluate predictors of CR in patients undergoing treatment with PCD or EUS-guided ethanol sclerotherapy. By analyzing a large, single-center cohort, we seek to identify factors that enhance treatment effectiveness and determine optimal timing for therapeutic intervention in patients with symptomatic hepatic cysts.

## METHODS

### Study design and patient selection

This retrospective cohort study was conducted at a tertiary referral center between January 2003 and December 2023. Consecutive patients who underwent interventional treatment for hepatic cysts were reviewed. Eligible cases included patients with unilocular or multilocular hepatic cysts who were treated with either PCD with ethanol retention therapy (PCD-ERT) or EUS-guided ethanol lavage (EL). Inclusion criteria were (1) symptomatic hepatic cysts, including abdominal pain, fullness, or early satiety; (2) radiologically confirmed cyst volume greater than 5 cm in maximal diameter or ≥100 mL in volume; and (3) a minimum follow-up period of 3 months with available imaging. Patients with polycystic liver disease or coexisting malignancy were excluded.

This study was approved by the Institutional Review Board of Asan Medical Center (IRB No. 2025-0019). The requirement for written informed consent was waived owing to the retrospective study design.

### Procedures

Treatment modality was selected based on cyst size, location, and feasibility. PCD-ERT was performed under ultrasound guidance using a 7–8.5 Fr catheter. After drainage, 99.9% ethanol was instilled. For cysts ≤200 mL, ethanol volume matched the aspirated volume; for larger cysts, ethanol was limited to 200 mL. The ethanol was retained for 20–40 min, with position changes every 5 min.

EUS-EL was performed by an experienced endosonographer (D.-W. Seo) using a linear-array echoendoscope (GF-UCT 260; Olympus Corporation, Tokyo, Japan) with a 19-gauge fine-needle aspiration needle under real-time ultrasound guidance. The needle was introduced into the cyst cavity, and cyst fluid was aspirated to the maximum possible volume while maintaining stable needle positioning. EL was subsequently performed by injecting 99.9% ethanol at a 1:1 volume ratio to the aspirated fluid, with a maximum ethanol volume of 200 mL regardless of aspirated volume. The ethanol was retained briefly within the cyst cavity and then completely aspirated to ensure full cyst evacuation before needle withdrawal. All procedures were performed under conscious sedation with intravenous midazolam and meperidine. Prophylactic third-generation cephalosporins were administered intravenously.

### Data collection

Baseline patient data were retrieved from electronic medical records and included age, sex, body mass index, and comorbidities such as diabetes mellitus, hypertension, hepatitis, smoking, and alcohol use. Cyst characteristics included maximal diameter (cm), calculated volume (mL), and location (right *vs.* left hepatic lobe). Laboratory values obtained before and after treatment included aspartate aminotransferase (AST), alanine aminotransferase (ALT), alkaline phosphatase (ALP), C-reactive protein (CRP), and white blood cell count (WBC). Imaging findings were independently reviewed by 2 radiologists blinded to clinical outcomes.

Treatment response was assessed by comparing cyst volume before and after the procedure using the last follow-up computed tomography (CT) scan. Cyst volume was calculated using the ellipsoidal formula (width × height × length × 0.523), based on 3-dimensional measurements obtained from CT imaging. Response was categorized as CR (<5% of baseline volume), partial resolution (5–25%), or persistent cyst (>25%).

### Follow-up evaluation

Post-procedural monitoring included laboratory tests (complete blood count, liver function tests, chemistry panel, and CRP and a plain abdominal radiograph on the day after the procedure to identify adverse events. All patients were observed for a minimum of 2 days with continued intravenous administration of prophylactic antibiotics. Follow-up evaluation with CT imaging and clinical assessment was performed at 6 months, 1 year, and yearly thereafter until cyst resolution, followed by biennial follow-up after CR. Additional visits were scheduled if symptoms or complications occurred.

### Statistical analysis

Continuous variables are presented as mean ± standard deviation for normally distributed data or median with interquartile range (IQR) for skewed data. Group comparisons were performed using the independent *t* test or the Mann–Whitney *U* test, as appropriate. Categorical variables are expressed as counts and percentages and compared using the chi-square test or Fisher’s exact test. To evaluate the predictive value of initial cyst volume for CR, receiver operating characteristic (ROC) curve analysis was performed. The optimal cutoff was determined using Youden’s index, and the area under the curve (AUC), sensitivity, specificity, and accuracy were calculated. Multivariable logistic regression analysis was conducted to identify independent predictors of CR. Variables with a *P* value <0.2 in univariate analysis were included in the multivariable model. Adjusted odds ratios (aORs) with 95% confidence intervals (CIs) were calculated. All statistical analyses were performed using R version 4.2.2 (R Foundation for Statistical Computing, Vienna, Austria), and a 2-sided *P* value <0.05 was considered statistically significant.

## RESULT

### Baseline characteristics and treatment outcome

A total of 307 patients with symptomatic hepatic cysts who underwent interventional treatment were included in the analysis. **Table 1** summarizes the baseline characteristics and clinical outcomes of the study population. The mean age was 68.3 ± 10.0 years, and the majority were female (78.5%). Comorbidities included hypertension (43.6%), diabetes mellitus (3.6%), and hepatitis (2.6%). Among the included patients, 109 (35.5%) underwent EUS-EL, while 198 (64.5%) received PCD-ERT.

**Table 1:** Baseline clinical and cyst characteristics.

Characteristic	All patients (*n* = 307)	EUS-EL (*n* = 109)	PCD-ERT (*n* = 198)	*P* value
**Patient characteristics**				
Age, yr, mean ± SD	68.28 ± 10.03	65.9 ± 10.1	69.6 ± 9.8	0.002
Female, *n* (%)	241 (78.5)	86 (78.9)	155 (78.3)	0.899
BMI, kg/m^2^, mean ± SD	24.42 ± 3.76	24.75 ± 3.53	24.25 ± 3.87	0.280
Diabetes mellitus	11 (3.6)	4 (3.7)	7 (3.5)	0.948
Hypertension	134 (43.6)	50 (45.9)	84 (42.4)	0.572
Hepatitis	8 (2.6)	3 (2.8)	5 (2.5)	0.894
Smoking	40 (13.0)	14 (12.8)	26 (13.1)	0.948
Alcohol use	80 (26.1)	28 (25.7)	52 (26.3)	0.907
**Cyst characteristics**				
Initial diameter (max), cm, mean ± SD	10.5 ± 4.7	7.7 ± 3.0	12.1 ± 4.7	<0.001
Initial cyst volume, mL, mean ± SD	623.16 ± 812.67	228.19 ± 239.33	840.58 ± 927.70	<0.001
Location, *n* (%)				<0.001
Right lobe	169 (55.0)	32 (29.4)	137 (69.2)	
Left lobe	138 (45.0)	77 (70.6)	61 (30.8)	
**Laboratory parameters**				
AST, U/L, mean ± SD	22.4 ± 8.4	21.2 ± 7.1	23.0 ± 9.0	0.046
ALT, U/L, mean ± SD	19.0 ± 11.0	16.9 ± 7.9	20.2 ± 12.2	0.010
ALP, U/L, mean ± SD	81.1 ± 47.5	72.9 ± 20.7	85.7 ± 56.7	0.022
GGT, U/L, mean ± SD	40.9 ± 121.5	20.3 ± 12.9	52.2 ± 149.9	0.005
WBC, ×10^3^/μL, mean ± SD	6.2 ± 1.9	6.1 ± 1.4	6.2 ± 2.1	0.639
CRP, mg/dL, mean ± SD	0.5 ± 1.6	0.1 ± 0.1	0.6 ± 2.0	0.062

ALP, alkaline phosphatase; ALT, alanine aminotransferase; AST, aspartate aminotransferase; BMI, body mass index; CRP, C-reactive protein; EUS-EL, EUS-guided ethanol lavage; GGT, gamma-glutamyl transferase; PCD-ERT, percutaneous catheter drainage with ethanol retention; SD, standard deviation; WBC, white blood cell.

*P* values compare EUS-EL *versus* PCD-ERT groups.

The mean initial cyst volume and diameter were 623 ± 817 mL and 10.5 ± 4.7 cm, respectively. EUS-EL patients had significantly smaller cysts than PCD-ERT patients (volume: 228 ± 239 mL *vs*. 840 ± 927 mL; diameter: 7.7 ± 3.0 cm *vs*. 12.1 ± 4.7 cm; both *P* < 0.001). The majority of cysts were located in the right hepatic lobe (55.0%). PCD-ERT patients were also significantly older (69.6 ± 9.8 *vs*. 65.9 ± 10.1 years, *P* = 0.002) and more likely to have right-lobe cysts (69.2% *vs*. 29.4%, *P* < 0.001) compared with the EUS-EL group.

After a mean follow-up of 24.7 ± 21.1 months, CR was achieved in 167 patients (54.4%), partial resolution in 97 (31.6%), and persistent cysts were observed in 43 (14.0%) (**Table 2**). Importantly, the CR rate was similar between treatment groups (EUS-EL 56.0% *vs*. PCD-ERT 53.5%, *P* = 0.678), indicating that treatment selection was appropriately matched to cyst characteristics rather than reflecting differential treatment efficacy.

**Table 2:** Treatment response and complete resolution rates.

Characteristic	All patients (*n* = 307)	EUS-EL (*n* = 109)	PCD-ERT (*n* = 198)	*P* value
Complete resolution, *n* (%)	167 (54.4)	61 (56.0)	106 (53.5)	0.678
Partial resolution, *n* (%)	97 (31.6)	40 (36.7)	57 (28.8)	
Persistent cyst, *n* (%)	43 (14.0)	8 (7.3)	35 (17.7)	
Follow-up duration, months, mean ± SD	24.7 ± 21.1	17.4 ± 15.3	28.7 ± 22.8	<0.001

EUS-EL, EUS-guided ethanol lavage; PCD-ERT, percutaneous catheter drainage with ethanol retention; SD, standard deviation.

*P* values compare EUS-EL *versus* PCD-ERT groups.

### Treatment response and volume changes over time

**Figure 1.**
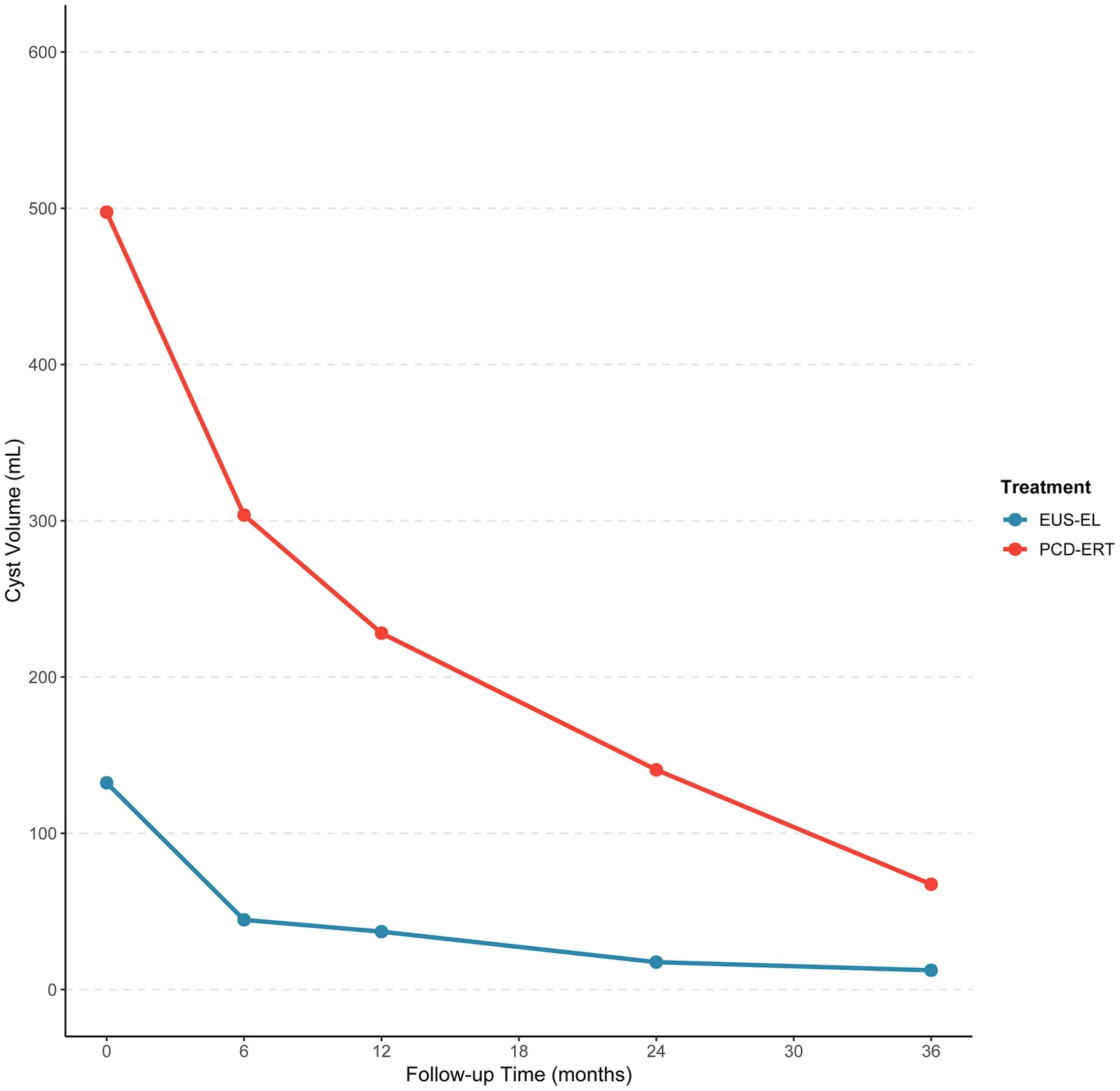
**Changes in cyst volume after therapy by treatment group.** Median cyst volumes over time for EUS-guided ethanol lavage therapy (EUS-EL, blue line) and percutaneous catheter drainage with ethanol retention therapy (PCD-ERT, red line).

Figure 1 illustrates the temporal changes in median cyst volume after intervention with either EUS-EL or PCD-ERT over a 36-month follow-up period. Initially, PCD-ERT patients had significantly larger median cyst volumes compared with EUS-EL patients (497 *vs*. 132 mL, *P* < 0.001). Both treatment modalities demonstrated substantial volume reduction, with the most pronounced decreases occurring within the first 6 months.

EUS-EL showed a more rapid initial response and consistent downward trend, with median volumes decreasing to 44 mL by 6 months and continuing to decline to 12 mL by 36 months, representing a 91% volume reduction. The PCD-ERT group showed a more gradual but sustained downward trend, ultimately achieving a median volume of 67 mL by 36 months, representing an 86% volume reduction.

### Comparison of complete resolution and noncomplete resolution groups

**Table 3** presents a comparison between patients who achieved CR and those who did not (non-CR). Patients who achieved CR had significantly smaller initial cyst volumes (484 ± 667 *vs*. 788 ± 933 mL, *P* = 0.002) and diameters (9.7 ± 4.4 *vs*. 11.6 ± 4.8 cm, *P* < 0.001) compared with non-CR patients. The proportion of small cysts (<200 mL) was significantly higher in the CR group (46.1% *vs*. 24.3%, *P <* 0.001), whereas large cysts (≥500 mL) were more common in the non-CR group (46.4% *vs*. 29.3%, *P* = 0.002).

**Table 3:** Comparison of baseline characteristics between complete resolution and noncomplete resolution groups.

Characteristics	CR (*n* = 167)	Non-CR (*n* = 40)	*P* value
Patient characteristics			
Age, yr, mean ± SD	69.1 ± 9.2	67.3 ± 10.9	0.089
Female, *n* (%)	127 (76.0)	114 (81.4)	0.254
BMI, kg/m^2^, mean ± SD	24.3 ± 3.3	24.6 ± 4.2	0.461
Diabetes mellitus	2 (1.2)	9 (6.4)	0.014
Hypertension	77 (46.1)	57 (40.7)	0.343
Hepatitis	4 (2.4)	4 (2.9)	0.801
Smoking	17 (10.2)	23 (16.4)	0.105
Alcohol use	46 (27.5)	34 (24.3)	0.517
Follow-up duration, months, mean ± SD	28.8 ± 21.2	19.8 ± 20.1	<0.001
Cyst characteristics			
Initial diameter (max), cm, mean ± SD	9.7 ± 4.4	11.6 ± 4.8	<0.001
Initial volume, mL, mean ± SD	484.3 ± 667.8	788.8 ± 933.0	0.002
Volume <200 mL, *n* (%)	77 (46.1)	34 (24.3)	<0.001
Volume ≥500 mL, *n* (%)	49 (29.3)	65 (46.4)	0.002
Location, *n* (%)			0.110
Right lobe	85 (50.9)	84 (60.0)	
Left lobe	82 (49.1)	56 (40.0)	
Treatment modality, *n* (%)			
EUS-EL	61 (36.5)	48 (34.3)	0.681
PCD-ERT	106 (63.5)	92 (65.7)	
Laboratory parameters			
AST >30 U/L, *n* (%)	10 (6.0)	21 (15.0)	0.008
ALT >30 U/L, *n* (%)	4 (2.4)	19 (13.6)	<0.001
CRP, mg/dL, mean ± SD	0.5 ± 1.9	0.4 ± 1.2	0.611
WBC, ×10^3^/μL, mean ± SD	6.1 ± 2.0	6.2 ± 1.8	0.652

ALP, alkaline phosphatase; ALT, alanine aminotransferase; AST, aspartate aminotransferase; BMI, body mass index; CR, complete resolution; CRP, C-reactive protein; EUS-EL, EUS-guided ethanol lavage; GGT, gamma-glutamyl transferase; non-CR includes partial resolution and persistent cyst; PCD-ERT, percutaneous catheter drainage with ethanol retention; SD, standard deviation; WBC, white blood cell.

The most notable clinical difference was the significantly lower prevalence of diabetes mellitus in the CR group (1.2% *vs*. 6.4%, *P* = 0.014). Other demographic factors, comorbidities, cyst location, and treatment modality distribution showed no significant differences between groups.

Regarding laboratory parameters, patients in the CR group had better liver function profiles. Elevated AST >30 U/L occurred in only 6.0% of CR patients *versus* 15.0% of non-CR patients (*P* = 0.008), and elevated ALT >30 U/L was found in 2.4% *versus* 13.6%, respectively (*P* < 0.001).

### Factors associated with complete resolution

Univariable and multivariable logistic regression analyses were performed to identify independent predictors of CR. In univariable analysis, initial cyst volume ≥233 mL was the strongest predictor of treatment failure (OR 0.32, 95% CI 0.20–0.52, *P* < 0.001), and diabetes mellitus was significantly associated with reduced CR (OR 0.18, 95% CI 0.04-0.83, *P* = 0.028).

**Figure 2.**
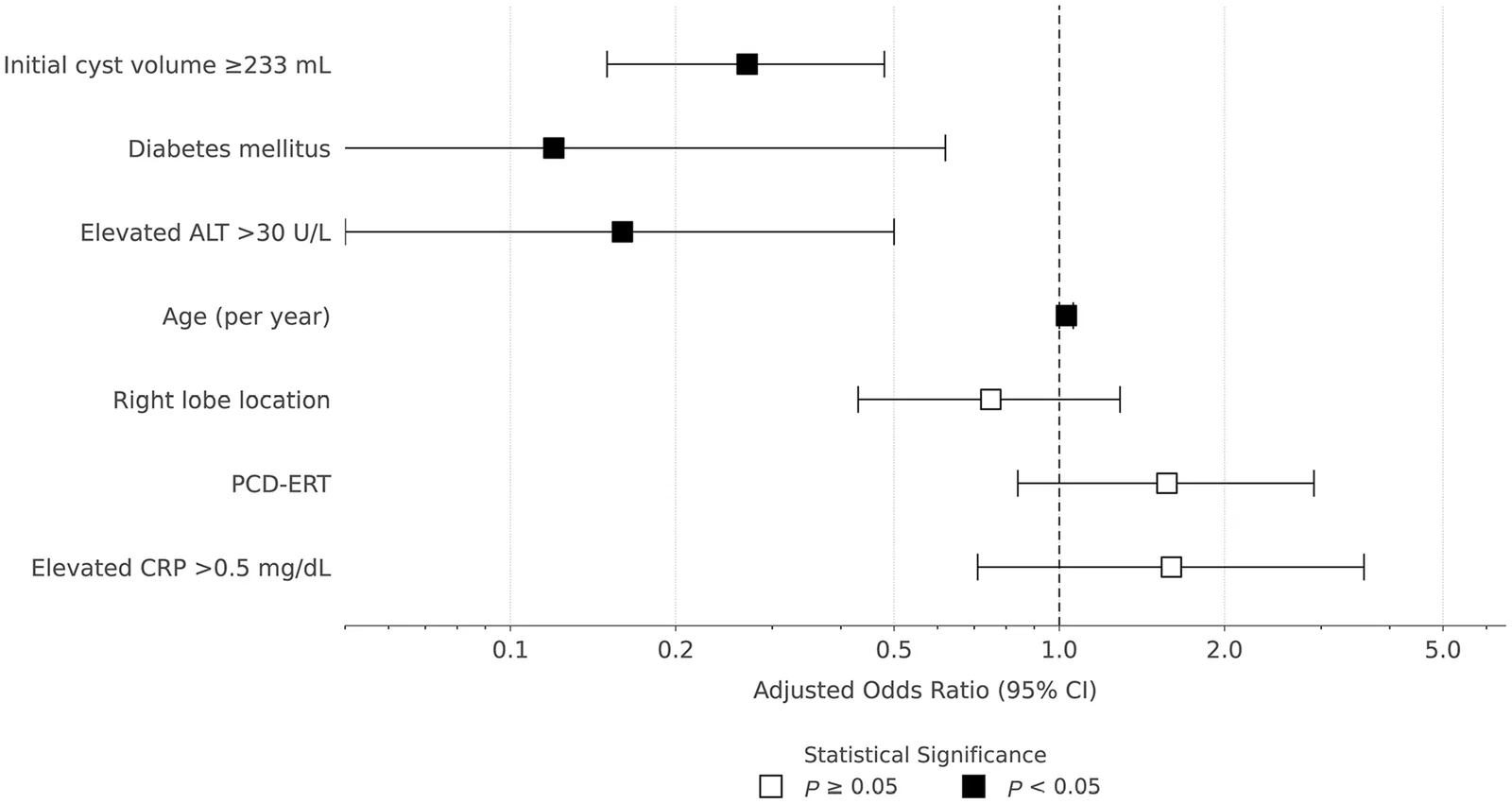
**Multivariable predictors of complete resolution (Forest plot).** Adjusted odds ratios (ORs) with 95% confidence intervals (CIs) from multivariable logistic regression analysis. Black squares indicate statistically significant associations (*P* < 0.05), while white squares represent nonsignificant associations. The vertical dashed line represents null effect (OR = 1.0). Variables to the left of the line are associated with decreased odds of complete resolution, while those to the right indicate increased odds. ALT, alanine aminotransferase; CRP, C-reactive protein.

Figure 2 shows the multivariable analysis results, revealing 4 independent predictors: initial cyst volume ≥233 mL (aOR 0.27, 95% CI 0.15–0.48, *P* < 0.001), diabetes mellitus (aOR 0.12, 95% CI 0.02–0.62, *P* = 0.011), elevated ALT >30 U/L (aOR 0.16, 95% CI 0.05–0.50, *P* = 0.002), and age per year (aOR 1.03, 95% CI 1.00–1.06, *P* = 0.023). Other variables, including PCD-ERT procedure, right-lobe location, and elevated CRP, did not achieve statistical significance. The estimate for diabetes mellitus should be interpreted with caution, as it was based on only 11 patients (2 in the CR group and 9 in the non-CR group). Complete regression results with all variables are provided in Supplementary Appendix Table A1, https://links.lww.com/ENUS/A408.

### Volume threshold analysis

**Figure 3.**
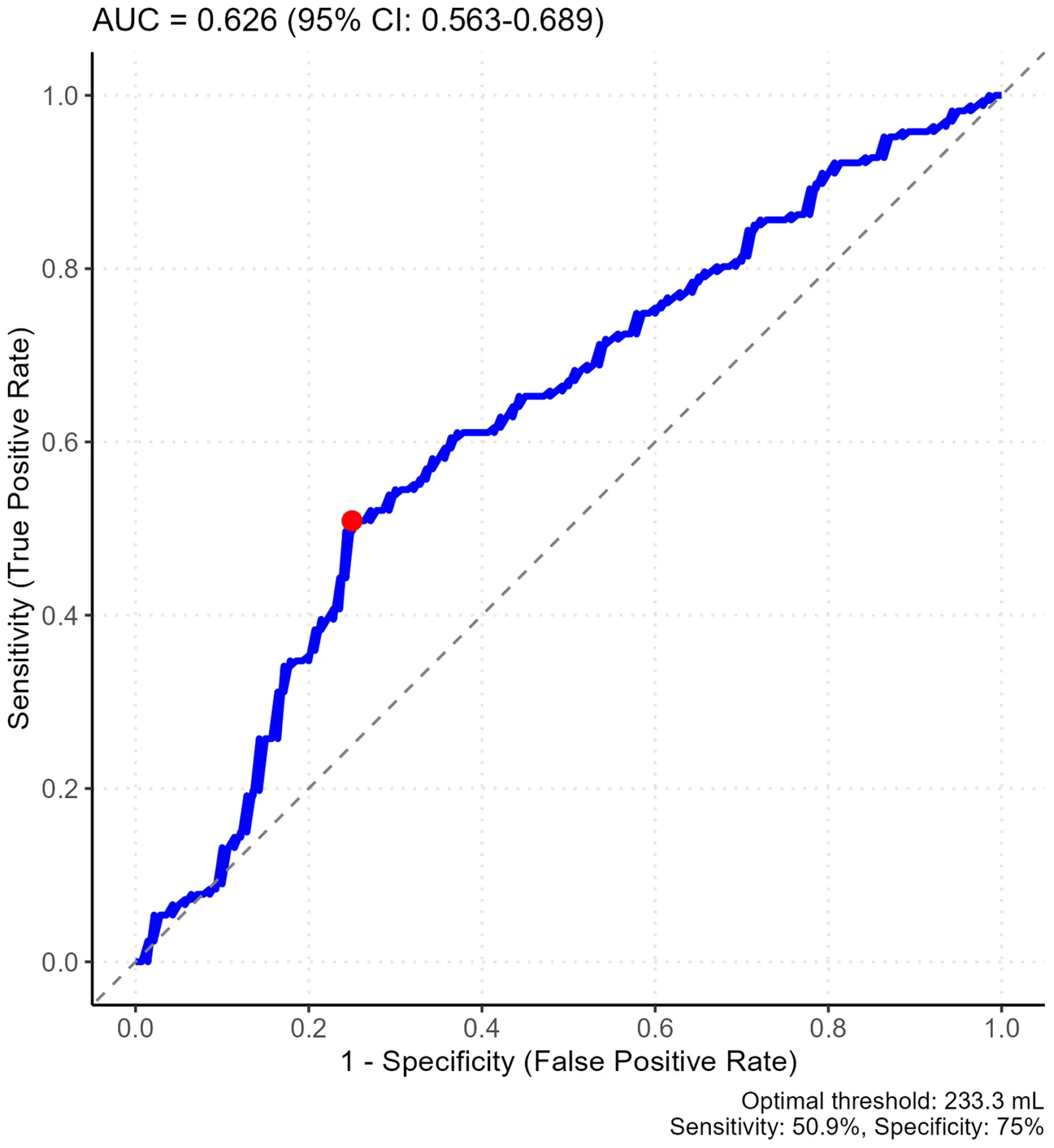
**Receiver operating characteristic (ROC) curve for initial cyst volume predicting complete resolution.** The ROC curve demonstrates the diagnostic performance of initial cyst volume as a predictor of complete resolution after ethanol sclerotherapy. The area under the curve (AUC) was 0.626 (95% confidence interval [CI] 0.56–0.69). The threshold identified by Youden’s index was 233 mL, providing 50.9% sensitivity and 75.0% specificity. The diagonal dashed line represents random prediction (AUC = 0.5).

To identify an initial cyst volume threshold that best discriminated CR from non-CR, we performed ROC curve analysis (Figure 3). The AUC was 0.626 (95% CI: 0.56–0.69), indicating fair discriminatory capacity for volume-based prediction of treatment success.

ROC analysis identified an initial cyst volume of 233 mL as the threshold with the highest Youden’s index (sensitivity 50.9%, specificity 75.0%, accuracy 61.6%). At this threshold, CR was achieved in 70.6% of cysts smaller than 233 mL compared with 44.1% of those ≥233 mL, resulting in a risk difference of 26.4% and a number needed to treat of 3.8.

Analysis of various volume thresholds revealed a consistent trend of decreasing CR rates with increasing cyst size. The highest CR rates were observed in cysts measuring 100–200 mL (69.4%), while CR rates declined progressively in larger cysts: 66.9% for 200–300 mL, 63.3% for 300–400 mL, and 61.1% for cysts >500 mL.

When ROC analysis was performed separately by treatment modality, both EUS-EL (AUC 0.617) and PCD-ERT (AUC 0.644) demonstrated similar discriminatory capacity for volume-based outcome prediction, with no statistically significant difference between curves (*P* = 0.692). This finding suggests that the volume threshold applies consistently across both treatment approaches.

## DISCUSSION

In this analysis of 307 patients with symptomatic hepatic cysts treated with ethanol sclerotherapy, cyst volume and diabetes mellitus were associated with CR. Our multivariable analysis established an initial cyst volume of 233 mL (approximately 7–8 cm in diameter) as a threshold for predicting CR, with smaller cysts demonstrating significantly higher success rates. These findings may help inform treatment decisions in symptomatic patients.

The volume-dependent treatment response observed in our study aligns with theoretical principles of sclerotherapy efficacy. Smaller cysts allow for more uniform ethanol distribution, higher sclerosant-to-fluid ratios, and better wall apposition after aspiration.^[14,15]^ Conversely, larger cysts often exhibit internal complexity such as septations or multilocularity, which may limit sclerosant contact and treatment efficacy.^[7,14]^ Additionally, larger cysts are more likely to contain debris that interferes with complete aspiration and uniform ethanol distribution.^[16]^ Our findings suggest that cyst size at the time of treatment may influence the likelihood of resolution. The CR rate declined from 70.6% in cysts <233 mL to 44.1% in those ≥233 mL, with larger cysts showing significantly lower odds of treatment success (aOR 0.27, 95% CI 0.15–0.48). Intervention before marked cyst enlargement may also reduce the procedural complexity and complications associated with very large cysts, including compression of adjacent structures, rupture, or secondary infection.

However, the 233 mL (7–8 cm diameter) threshold should be interpreted as a guidance tool rather than a rigid treatment criterion. Importantly, this threshold does not imply that all cysts smaller than this volume require immediate treatment, nor that all cysts approaching this size mandate intervention. The threshold represents a point where treatment efficacy begins to decline significantly, making it most valuable for counseling symptomatic patients about treatment timing and for avoiding unnecessary delays in appropriate candidates. Treatment decisions should always be individualized based on symptom severity, impact on quality of life, patient comorbidities, procedural risk, and patient preferences. For asymptomatic or minimally symptomatic patients, careful observation may remain appropriate regardless of cyst size.

Diabetes mellitus emerged as an independent negative predictor of treatment success (aOR 0.12, 95% CI 0.02–0.62). Only 11 patients in our cohort had diabetes (2 in the CR group and 9 in the non-CR group), so the precision of this estimate is limited, and the association warrants confirmation in larger cohorts. The pathophysiological mechanisms underlying the reduced treatment response in diabetic patients may include chronic low-grade inflammation, impaired epithelial repair, and diminished microvascular integrity, all of which can interfere with effective ethanol-induced sclerosis.^[17,18]^

Our analysis revealed no significant difference in CR rates between EUS-EL and PCD-ERT (56.0% *vs*. 53.5%, *P* = 0.678), and treatment modality was not significantly associated with outcomes in multivariable analysis (aOR 1.57, 95% CI 0.84–2.91, *P* = 0.156). This finding suggests that treatment selection was appropriately matched to anatomical and clinical factors rather than reflecting inherent differences in treatment efficacy. In our cohort, EUS-EL was preferentially used for left-lobe cysts and PCD-ERT for right-lobe cysts, reflecting differences in anatomical accessibility. The EUS-guided approach allows direct visualization and complete aspiration of left-lobe cysts adjacent to the stomach, often without the need for catheter placement, whereas PCD-ERT enables prolonged drainage and is more suitable for large, right-lobe cysts.^[19]^ Furthermore, the similar ROC performance between the two modalities (EUS-EL AUC 0.617 *vs*. PCD-ERT AUC 0.644, *P* = 0.692) indicates that the 233 mL volume threshold performs consistently regardless of treatment modality, suggesting that cyst volume is a relevant consideration across both approaches.

Several limitations should be acknowledged. The retrospective, single-center design may limit generalizability. The discriminatory performance of cyst volume for predicting CR was modest (AUC 0.626), indicating that factors beyond volume contribute meaningfully to treatment outcome; the 233 mL threshold should therefore be used as a reference point rather than a stand-alone decision rule. In addition, the diabetes mellitus subgroup was small (*n* = 11), and the corresponding estimate should be confirmed in larger cohorts.

Future research should focus on prospective, multicenter validation of the 233 mL threshold and on confirming the observed association between diabetes mellitus and treatment outcome in larger cohorts. Integrated prediction models incorporating multiple clinical factors could further refine patient selection, while cost-effectiveness analyses comparing treatment strategies would provide valuable economic evidence for clinical decision-making.

In conclusion, initial cyst volume is an important factor influencing treatment outcome after ethanol sclerotherapy. The 233 mL threshold may serve as a useful reference for the timing of intervention. Treatment decisions should integrate cyst size with symptoms and patient-specific factors.

## Supplementary Information

Supplemental digital content is available for this article. Direct URL citations are provided in the HTML and PDF versions of this article on the journal’s Web site (www.eusjournal.com).

## Ethical Statement

This study was approved by the Institutional Review Board of Asan Medical Center (IRB No. 2025-0019) and was conducted in accordance with the Declaration of Helsinki. The requirement for written informed consent was waived by the Institutional Review Board owing to the retrospective nature of the study.

## Conflicts of Interest

Dong-Wan Seo is an Editorial Board Member of the journal. The article was subjected to the standard procedures of the journal, with a review process independent of the editor and his research group. The other authors declare no conflicts of interest.

## Author Contributions

Y. Lee: Study design, data interpretation, manuscript drafting, and revision. D.-W. Seo: Conceptualization, supervision, and critical revision of the manuscript. S.H. Cho, G. Huh, D. Oh, and T.J. Song: Review and approval of the final manuscript. All authors reviewed and approved the final version of the manuscript.

## Use of Large Language Models, AI and Machine Learning Tools

None declared.

## Data Availability Statement

The data underlying this article are available from the corresponding author upon reasonable request.
